# Supplementary material for: Use of Large-Scale Genomics to Identify the Role of Animals and Foods as Potential Sources of Extraintestinal Pathogenic Escherichia coli That Cause Human Illness
Source: Foods. 2022 Jul 3;11(13):1975. doi: 10.3390/foods11131975 (PMC9265580; doi:10.3390/foods11131975)
Supplement: Supplementary file 1 [file foods-11-01975-s001.zip › Supplemental_Figures.pdf]

Sequence Type Subdivisions of non-IPEC Virulence Groups Containing ≥ 5 Isolates from Humans

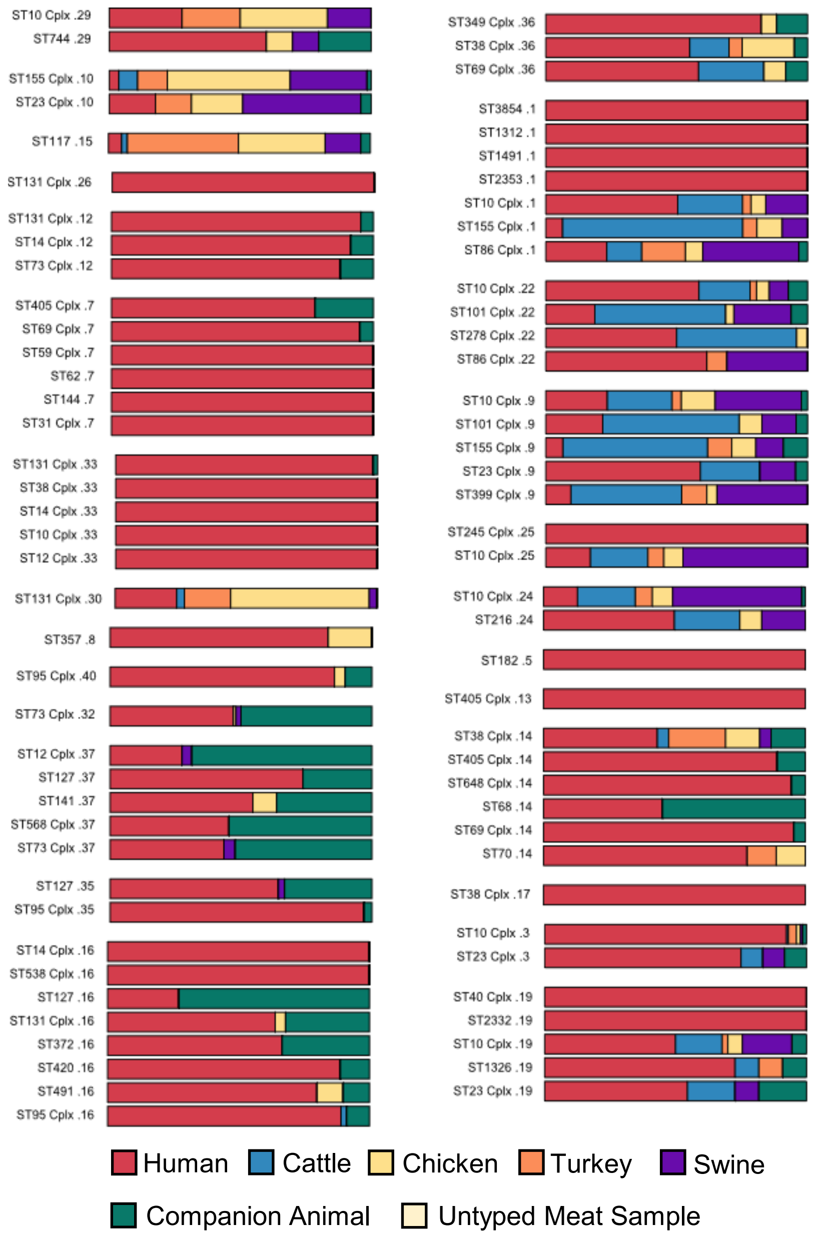

Supplemental Figure S1. A comparison of sequence type/ virulence group combinations shows how the human proportion of isolates from a given sequence type differs by virulence group. Label format is ST followed by virulence group (e.g. ST10 Cplx .29 is the ST10 Cplx from virulence group 29)

Composition of Virulence Groups by Phylotype, Organization, Isolation Source and Sequence Type

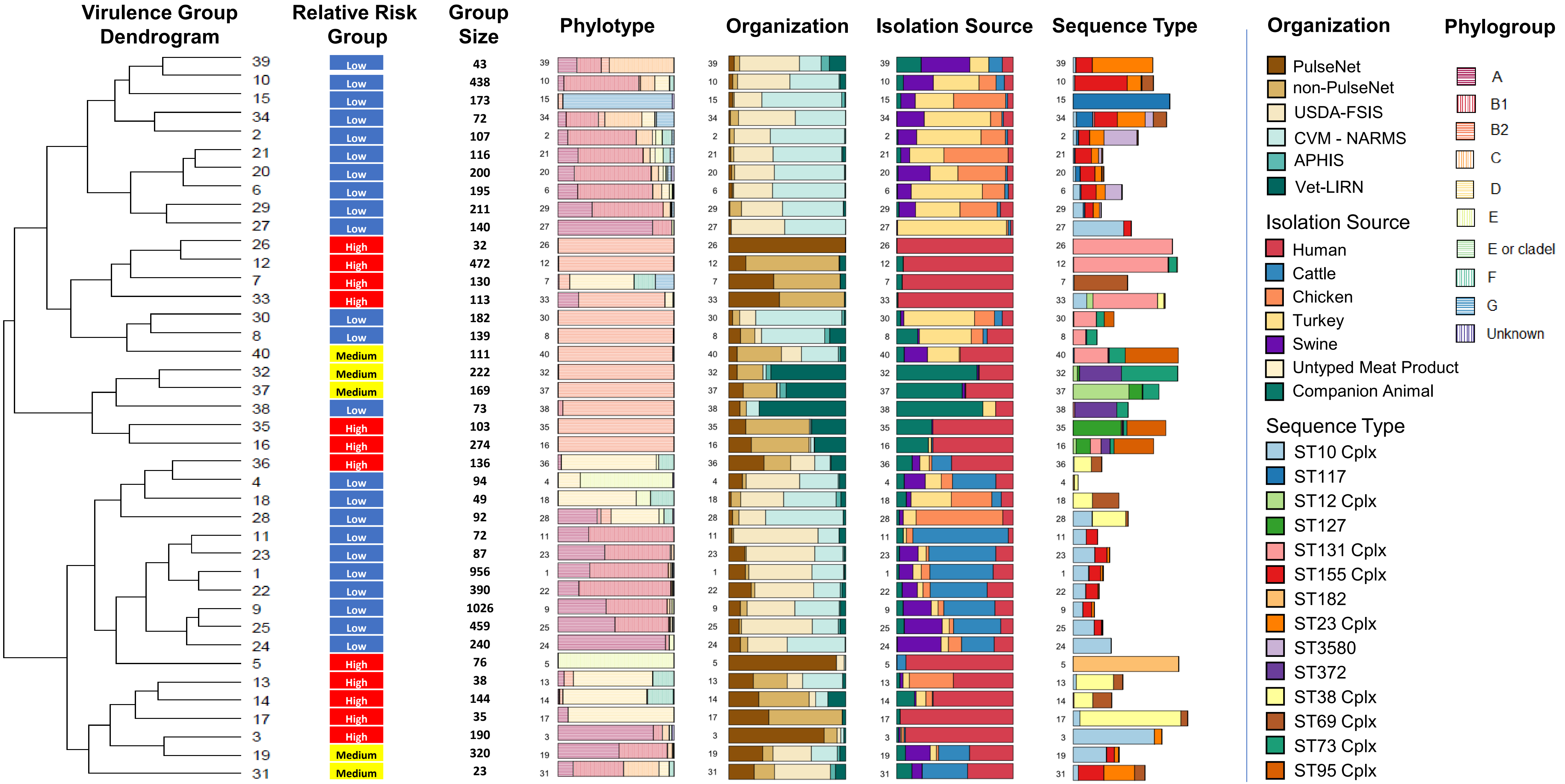

Supplemental Figure S2. An overview of virulence groups organized by: virulence gene profile dendrogram; relative risk group and size; and composition by phylogenetic group, contributing organization, isolation source and sequence type. Corresponding data can be found in Supplemental Tables 1-5. Animal isolation sources are the combined results from all contributing organizations
