# Supplementary material for: Use of Large-Scale Genomics to Identify the Role of Animals and Foods as Potential Sources of Extraintestinal Pathogenic Escherichia coli That Cause Human Illness
Source: Foods. 2022 Jul 3;11(13):1975. doi: 10.3390/foods11131975 (PMC9265580; doi:10.3390/foods11131975)
Supplement: Supplementary file 1 [file foods-11-01975-s001.zip › Supplemental_Tables.pdf]

Supplemental Table S1. Virulence Group Definitions

Virulence Group	Virulence Genes
**39**	*espX1, fdeC, fyuA, iroE, iroN, irp2, iss, iucA, iucC, iutA, lpfA, mchF, ompT, papA, papC, sitA, traT, ybtP, ybtQ, hlyF, gad, cvaC, hra, etsC, papH*
**10**	*espX1, fdeC, fyuA, iroE, iroN, irp2, iss, iucA, iucC, iutA, lpfA, mchF, ompT, sitA, traT, ybtP, ybtQ, hlyF, gad, cvaC, etsC*
**15**	*chuA, fdeC, fyuA, iroE, iroN, irp2, iss, iucA, iucC, iutA, lpfA, ompT, sitA, sslE, traT, ybtP, ybtQ, vat, hlyF, etsC, ireA*
**34**	*espX1, fdeC, fyuA, iroE, iroN, irp2, iss, iucA, iucC, iutA, lpfA, mchF, ompT, papC, papF, papG, sitA, traT, ybtP, ybtQ, hlyF, papE, gad, cvaC, etsC, tsh*
**2**	*espX1, fdeC, iroE, iroN, iss, iucA, iucC, iutA, lpfA, mchF, ompT, papA, papC, papF, papG, sitA, traT, hlyF, papE, gad, etsC*
**21**	*cib, espX1, fdeC, iroE, iroN, iss, iucA, iucC, iutA, lpfA, mchF, ompT, sitA, traT, hlyF, gad, cvaC, etsC, tsh*
**20**	*espX1, fdeC, iroE, iroN, iss, iucA, iucC, iutA, lpfA, mchF, ompT, sitA, traT, hlyF, gad, cvaC, etsC*
**6**	*cma, espX1, fdeC, iroE, iroN, iss, iucA, iucC, iutA, lpfA, mchF, ompT, sitA, traT, hlyF, gad, cvaC, etsC, tsh, cba*
**29**	*cma, espX1, fdeC, iroE, iroN, iss, lpfA, ompT, sitA, traT, hlyF, gad, cvaC*
**27**	*fdeC, iroE, iroN, iss, mchF, ompT, traT, hlyF, gad, astA, etsC*
**26**	*chuA, fdeC, fyuA, iha, irp2, iss, iucA, iucB, iucC, iucD, iutA, ompT, papA, sat, sitA, traT, usp, ybtP, ybtQ, yfcV, kpsE, gad, kpsM*
**12**	*chuA, fdeC, fyuA, iha, irp2, iss, iucA, iucC, iutA, ompT, papA, sat, sitA, traT, usp, ybtP, ybtQ, yfcV, kpsE, kpsM*
**7**	*chuA, eilA, espX1, fdeC, fyuA, iha, irp2, iucA, iucC, iutA, lpfA, ompT, papA, papC, papF, papG, sat, sitA, traT, ybtP, ybtQ, kpsE, gad, kpsM, papH, air*
**33**	*afaA, afaB, afaC, afaD, chuA, fdeC, fyuA, iha, irp2, iss, iucA, iucC, iutA, ompT, papA, sat, sitA, traT, usp, ybtP, ybtQ, yfcV, nfaE, kpsE, gad, kpsM*
**30**	*chuA, fdeC, fyuA, iroE, iroN, irp2, iss, iucA, iucC, iutA, mchF, ompT, sitA, sslE, traT, usp, ybtP, ybtQ, yfcV, vat, hlyF, kpsE, cvaC, etsC, kpsM, ibeA, tsh*
**8**	*chuA, fdeC, fyuA, iroE, iroN, irp2, iss, ompT, sitA, sslE, traT, usp, ybtP, ybtQ, yfcV, vat, hlyF, kpsE, gad, ibeA*
**40**	*chuA, fdeC, fyuA, iroE, iroN, irp2, iss, iucA, iucC, iutA, mchF, ompT, papA, papC, papF, papG, sitA, sslE, traT, usp, ybtP, ybtQ, yfcV, hlyF, kpsE, papE, gad, cvaC, etsC, kpsM*
**32**	*chuA, fdeC, fyuA, iroE, iroN, irp2, iss, mchB, mchF, ompT, papA, papC, papF, papG, sitA, usp, ybtP, ybtQ, yfcV, mchC, vat, mcmA, gad, cea, focI, hlyA.alpha, hra, sfaF, focG, cnf1, papH, focCsfaE*
**37**	*chuA, fdeC, fyuA, iroE, iroN, irp2, iss, mchB, mchF, ompT, papA, papC, papF, papG, sitA, usp, ybtP, ybtQ, yfcV, mchC, vat, mcmA, kpsE, gad, cea, hlyA.alpha, hra, sfaD, sfaF, kpsM, cnf1, clbB, focC, tcpC*
**38**	*chuA, fdeC, fyuA, iroE, iroN, irp2, iss, lpfA, mchB, mchF, ompT, sitA, usp, ybtP, ybtQ, yfcV, mchC, vat, mcmA, gad, cea, focI, sfaF, focG, ibeA*
**35**	*chuA, fdeC, fyuA, iroE, iroN, irp2, iss, ompT, papA, papC, papF, papG, sitA, sslE, traT, usp, ybtP, ybtQ, yfcV, vat, kpsE, gad, hlyA.alpha, hra, sfaD, sfaF, kpsM, cnf1, clbB, sfaS*
**16**	*chuA, fdeC, fyuA, irp2, iss, ompT, sitA, sslE, traT, usp, ybtP, ybtQ, yfcV, vat, kpsE, gad, kpsM*
**36**	*chuA, eilA, espX1, fdeC, kpsE, gad, kpsM, air*
**4**	*chuA, eilA, espX1, fdeC, iss, lpfA1, gad, air*
**18**	*chuA, cma, eilA, espX1, fdeC, iroE, iroN, iss, ompT, sitA, traT, hlyF, gad, cvaC, air*
**28**	*cma, espX1, fdeC, iha, iss, iucA, iucC, iutA, ompT, sitA, traT, kpsE, gad, astA, hra, kpsM*
**11**	*fdeC, iss, lpfA, ompT, traT, cia, gad, hlyA.alpha*
**23**	*espX1, fdeC, ompT, traT, gad, astA*
**1**	*espX1, fdeC, iss, lpfA, ompT, traT, gad*
**22**	*espX1, fdeC, iss, lpfA, ompT, gad*
**9**	*espX1, fdeC, lpfA, gad*
**25**	*fdeC, gad*
**24**	*espX1, gad*
**5**	*chuA, eilA, espX1, fdeC, iss, lpfA, lpfA1, gad, astA, capU, sta1*

**13**	*chuA, eilA, espX1, fdeC, fyuA, irp2, iucA, iucC, iutA, sitA, traT, ybtP, ybtQ, kpsE, gad, hra, kpsM, air*
**14**	*chuA, eilA, espX1, fdeC, fyuA, irp2, ybtP, ybtQ, kpsE, gad, kpsM, air*
**17**	*afaA, afaB, afaC, afaD, chuA, eilA, espX1, fdeC, fyuA, irp2, iss, ybtP, ybtQ, nfaE, kpsE, gad, kpsM, air*
**3**	*espX1, fdeC, fyuA, iha, irp2, iucA, iucC, iutA, traT, ybtP, ybtQ, gad*
**19**	*espX1, fdeC, fyuA, irp2, ybtP, ybtQ, gad*
**31**	*afaA, afaB, afaC, afaD, fdeC, iss, iucA, iucC, iutA, lpfA, ompT, traT, gad, afaE8, bmaE*

Supplemental Table S2. Isolation Source Composition of Virulence Groups

VG	Human	Cattle	Chicken	Turkey	Swine	Untyped Meat Sample	Companion Animal	Isolates	Relative Risk Group
**39**	9.3	14.0	0.0	16.3	44.2	0.0	16.3	43	L
**10**	7.5	7.1	14.8	39.3	25.8	0.5	5.0	438	L
**15**	4.6	1.7	46.2	32.9	12.1	1.7	0.6	173	L
**34**	8.3	1.4	9.7	56.9	23.6	0.0	0.0	72	L
**2**	4.7	1.9	20.6	55.1	15.9	1.9	0.0	107	L
**21**	4.3	0.0	56.0	29.3	7.8	0.0	2.6	116	L
**20**	5.0	1.5	41.0	23.5	27.5	1.0	0.5	200	L
**6**	4.1	3.1	19.0	60.5	11.8	1.0	0.5	195	L
**29**	10.9	2.8	31.8	37.9	14.2	0.5	1.9	211	L
**27**	2.1	2.1	1.4	93.6	0.7	0.0	0.0	140	L
**26**	100.0	0.0	0.0	0.0	0.0	0.0	0.0	32	H
**12**	94.3	0.0	0.0	0.2	0.0	0.0	5.5	472	H
**7**	95.4	0.0	0.0	0.0	0.0	0.0	4.6	130	H
**33**	99.1	0.0	0.0	0.0	0.0	0.0	0.9	113	H
**30**	9.3	6.6	18.1	61.5	2.7	0.5	1.1	182	L
**8**	22.3	4.3	11.5	44.6	1.4	0.0	15.8	139	L
**40**	45.0	0.0	2.7	27.0	19.8	0.9	4.5	111	M
**32**	29.3	0.0	0.0	0.5	1.4	0.0	68.9	222	M
**37**	40.8	0.0	0.0	0.6	2.4	0.0	56.2	169	M
**38**	15.1	0.0	0.0	11.0	0.0	0.0	74.0	73	L
**35**	68.9	0.0	0.0	0.0	1.0	0.0	30.1	103	H
**16**	68.6	1.5	0.4	2.6	0.0	0.4	26.6	274	H
**36**	52.9	16.9	2.2	8.8	6.6	0.0	12.5	136	H
**4**	14.9	37.2	9.6	13.8	18.1	0.0	6.4	94	L
**18**	10.2	8.2	36.7	34.7	6.1	0.0	4.1	49	L
**28**	8.7	0.0	72.8	10.9	3.3	2.2	2.2	92	L
**11**	4.2	81.9	5.6	2.8	0.0	0.0	5.6	72	L
**23**	14.9	57.5	2.3	6.9	17.2	0.0	1.1	87	L
**1**	17.2	54.4	7.3	7.1	11.9	0.3	1.8	956	L
**22**	22.3	49.0	6.4	4.9	13.8	0.0	3.6	390	L
**9**	15.7	43.9	5.0	5.8	24.3	0.1	5.3	1026	L
**25**	10.7	40.7	3.7	6.1	32.5	0.0	6.3	459	L
**24**	16.3	28.3	11.3	6.3	37.5	0.4	0.0	240	L
**5**	92.1	7.9	0.0	0.0	0.0	0.0	0.0	76	H
**13**	50.0	0.0	36.8	5.3	2.6	2.6	2.6	38	H
**14**	68.8	0.7	5.6	8.3	1.4	0.0	15.3	144	H
**17**	97.1	0.0	0.0	0.0	0.0	0.0	2.9	35	H
**3**	92.6	1.1	2.1	1.1	1.6	0.0	1.6	190	H
**19**	37.5	26.9	1.9	5.3	21.9	0.0	6.6	320	M
**31**	39.1	43.5	0.0	0.0	8.7	0.0	8.7	23	M

Values for isolation source presented as percentages, VG column header describes the Virulence Group and animal isolation sources are the combined results from all contributing organizations.

Supplemental Table S3. Contributing Data Source Composition of Virulence Groups

VG	CDC-PulseNet	non-PulseNet	FSIS NARMS	CVM NARMS	APHIS	Vet-LIRN	Isolates	Relative Risk Group
**39**	4.7	4.7	51.2	18.6	7.0	14.0	43	L
**10**	3.4	4.1	44.7	42.0	0.9	4.8	438	L
**15**	3.5	1.2	23.7	68.2	2.9	0.6	173	L
**34**	1.4	6.9	48.6	43.1	0.0	0.0	72	L
**2**	0.9	3.7	30.8	63.6	0.9	0.0	107	L
**21**	1.7	2.6	33.6	58.6	0.9	2.6	116	L
**20**	2.5	2.5	34.0	60.0	0.5	0.5	200	L
**6**	3.1	1.0	33.3	62.1	0.0	0.5	195	L
**29**	0.9	10.0	35.1	52.1	0.0	1.9	211	L
**27**	2.1	0.0	45.7	52.1	0.0	0.0	140	L
**26**	100.0	0.0	0.0	0.0	0.0	0.0	32	H
**12**	14.6	79.7	0.2	0.0	0.4	5.1	472	H
**7**	38.5	56.9	0.0	0.0	0.0	4.6	130	H
**33**	43.4	55.8	0.0	0.0	0.0	0.9	113	H
**30**	3.3	6.0	13.7	73.6	2.2	1.1	182	L
**8**	10.1	12.2	5.8	54.0	4.3	13.7	139	L
**40**	7.2	37.8	17.1	31.5	1.8	4.5	111	M
**32**	7.2	22.1	0.0	2.7	4.1	64.0	222	M
**37**	12.4	28.4	0.6	2.4	5.3	50.9	169	M
**38**	9.6	5.5	0.0	11.0	0.0	74.0	73	L
**35**	14.6	54.4	0.0	1.0	1.0	29.1	103	H
**16**	19.3	49.3	1.5	2.9	0.4	26.6	274	H
**36**	30.1	22.8	20.6	13.2	0.7	12.5	136	H
**4**	11.7	3.2	45.7	33.0	1.1	5.3	94	L
**18**	2.0	8.2	36.7	44.9	4.1	4.1	49	L
**28**	3.3	5.4	22.8	66.3	0.0	2.2	92	L
**11**	2.8	1.4	72.2	18.1	0.0	5.6	72	L
**23**	13.8	1.1	58.6	24.1	1.1	1.1	87	L
**1**	14.2	2.9	54.0	27.1	0.6	1.2	956	L
**22**	19.2	3.1	50.3	22.8	1.5	3.1	390	L
**9**	9.9	5.8	40.8	38.0	1.0	4.5	1026	L
**25**	8.9	2.0	60.6	22.2	1.5	4.8	459	L
**24**	10.0	6.3	33.8	49.6	0.0	0.4	240	L
**5**	92.1	0.0	6.6	1.3	0.0	0.0	76	H
**13**	21.1	28.9	13.2	34.2	0.0	2.6	38	H
**14**	25.7	43.1	5.6	10.4	0.0	15.3	144	H
**17**	34.3	62.9	0.0	0.0	0.0	2.9	35	H
**3**	81.6	11.1	3.2	2.6	0.0	1.6	190	H
**19**	29.1	8.8	32.8	22.2	2.2	5.0	320	M
**31**	21.7	17.4	47.8	0.0	4.3	8.7	23	M

Values for contributing data source presented as percentages, VG column header describes the Virulence Group.

Supplemental Table S4. Phylogenetic Group Composition of Virulence Groups

VG	A	B1	B2	C	D	E	E or cladeI	F	G	Unknown	Relative Risk Group
**39**	16.3	20.9	7.0	55.8	0.0	0.0	0.0	0.0	0.0	0.0	L
**10**	5.0	64.6	1.4	12.3	12.6	0.2	0.0	3.9	0.0	0.0	L
**15**	0.0	0.0	4.0	0.0	0.0	0.0	0.0	0.0	94.2	1.7	L
**34**	6.9	27.8	5.6	31.9	11.1	0.0	0.0	1.4	15.3	0.0	L
**2**	8.4	58.9	0.0	14.0	2.8	5.6	0.0	8.4	1.9	0.0	L
**21**	17.2	56.0	0.0	6.0	4.3	6.9	0.0	6.0	3.4	0.0	L
**20**	14.0	66.0	0.5	6.0	4.0	2.5	0.0	1.5	3.0	2.5	L
**6**	16.9	64.6	0.0	7.7	6.7	2.6	0.0	0.5	0.5	0.5	L
**29**	29.4	61.1	0.0	6.2	0.5	0.5	0.0	0.5	1.9	0.0	L
**27**	81.4	16.4	0.0	0.0	0.0	2.1	0.0	0.0	0.0	0.0	L
**26**	0.0	0.0	100.0	0.0	0.0	0.0	0.0	0.0	0.0	0.0	H
**12**	0.4	0.0	99.2	0.0	0.4	0.0	0.0	0.0	0.0	0.0	H
**7**	0.8	0.0	9.2	0.0	55.4	0.0	0.0	18.5	16.2	0.0	H
**33**	17.7	0.0	74.3	0.0	7.1	0.0	0.0	0.9	0.0	0.0	H
**30**	0.0	0.0	100.0	0.0	0.0	0.0	0.0	0.0	0.0	0.0	L
**8**	0.0	0.0	99.3	0.0	0.7	0.0	0.0	0.0	0.0	0.0	L
**40**	0.0	0.0	99.1	0.0	0.0	0.0	0.0	0.0	0.9	0.0	M
**32**	0.0	0.5	99.5	0.0	0.0	0.0	0.0	0.0	0.0	0.0	M
**37**	0.0	0.0	100.0	0.0	0.0	0.0	0.0	0.0	0.0	0.0	M
**38**	0.0	4.1	95.9	0.0	0.0	0.0	0.0	0.0	0.0	0.0	L
**35**	0.0	0.0	100.0	0.0	0.0	0.0	0.0	0.0	0.0	0.0	H
**16**	0.0	0.0	100.0	0.0	0.0	0.0	0.0	0.0	0.0	0.0	H
**36**	2.9	0.0	0.0	0.0	81.6	2.2	0.0	13.2	0.0	0.0	H
**4**	0.0	0.0	0.0	0.0	19.1	79.8	0.0	0.0	0.0	1.1	L
**18**	0.0	0.0	0.0	0.0	67.3	12.2	0.0	20.4	0.0	0.0	L
**28**	33.7	3.3	8.7	0.0	41.3	4.3	0.0	7.6	0.0	1.1	L
**11**	26.4	73.6	0.0	0.0	0.0	0.0	0.0	0.0	0.0	0.0	L
**23**	40.2	56.3	1.1	2.3	0.0	0.0	0.0	0.0	0.0	0.0	L
**1**	27.3	67.7	0.0	1.9	1.2	0.6	0.0	0.2	0.8	0.3	L
**22**	17.9	79.2	0.0	1.0	0.8	0.3	0.0	0.0	0.8	0.0	L
**9**	41.4	52.6	0.0	2.2	1.9	1.7	0.0	0.1	0.0	0.1	L
**25**	49.0	46.2	0.9	0.7	1.7	0.0	0.0	1.1	0.2	0.2	L
**24**	92.5	3.3	0.0	0.0	0.0	4.2	0.0	0.0	0.0	0.0	L
**5**	0.0	0.0	0.0	0.0	0.0	100.0	0.0	0.0	0.0	0.0	H
**13**	5.3	0.0	7.9	0.0	68.4	0.0	0.0	18.4	0.0	0.0	H
**14**	0.7	0.7	2.8	0.0	72.2	0.7	0.0	22.2	0.7	0.0	H
**17**	8.6	0.0	0.0	0.0	91.4	0.0	0.0	0.0	0.0	0.0	H
**3**	82.1	7.9	0.0	5.8	0.0	0.0	2.1	0.0	0.0	2.1	H
**19**	52.5	41.6	0.3	4.1	0.6	0.9	0.0	0.0	0.0	0.0	M
**31**	13.0	43.5	0.0	30.4	8.7	0.0	0.0	4.3	0.0	0.0	M

Values for phylogenetic group presented as percentages, VG column header describes the Virulence Group.

Supplemental Table S5. Sequence Type Composition of Virulence Groups

VG	ST3580	ST372	ST127	ST155 Cplx	ST95 Cplx	ST69 Cplx	ST12 Cplx	ST73 Cplx	ST23 Cplx	ST10 Cplx	ST117	ST131 Cplx	ST38 Cplx	ST182	ST-Other*	Relative Risk Group
**39**	0.0	0.0	0.0	14.0	0.0	0.0	0.0	0.0	51.2	2.3	0.0	0.0	0.0	0.0	32.6	L
**10**	0.5	0.0	0.0	44.5	0.0	9.6	0.0	0.0	11.6	1.4	0.0	0.0	0.5	0.0	32.0	L
**15**	0.0	0.0	0.0	0.0	0.0	0.0	0.0	0.0	0.0	0.0	82.1	0.0	0.0	0.0	17.9	L
**34**	6.9	0.0	0.0	19.4	0.0	11.1	0.0	0.0	23.6	2.8	13.9	1.4	0.0	0.0	20.8	L
**2**	28.0	0.0	0.0	9.3	0.0	0.9	0.0	0.0	12.1	2.8	1.9	0.0	0.0	0.0	44.9	L
**21**	2.6	0.0	0.0	13.8	0.0	0.9	0.0	0.0	6.0	0.9	0.9	0.0	0.0	0.0	75.0	L
**20**	0.5	0.0	0.0	12.5	0.0	1.5	0.0	0.0	5.5	2.0	4.0	0.0	0.0	0.0	74.0	L
**6**	13.8	0.0	0.0	12.8	0.0	0.0	0.0	0.0	7.7	5.6	1.0	0.0	0.5	0.0	58.5	L
**29**	1.4	0.0	0.0	7.1	0.0	0.0	0.0	0.0	5.2	8.5	1.4	0.0	0.0	0.0	76.3	L
**27**	0.0	0.0	0.0	6.4	0.0	0.0	0.0	0.0	0.0	42.9	0.0	0.0	0.0	0.0	50.7	L
**26**	0.0	0.0	0.0	0.0	0.0	0.0	0.0	0.0	0.0	0.0	0.0	84.4	0.0	0.0	15.6	H
**12**	0.0	0.0	0.0	0.0	0.6	0.0	0.2	6.8	0.0	0.2	0.0	80.3	0.4	0.0	11.4	H
**7**	0.0	0.0	0.0	0.0	0.0	45.4	0.0	0.0	0.0	0.8	0.0	0.0	0.0	0.0	53.8	H
**33**	0.0	0.0	0.0	0.0	0.0	0.9	5.3	0.0	0.0	11.5	0.0	54.9	5.3	0.0	22.1	H
**30**	0.0	0.0	0.0	0.0	8.2	0.0	0.5	6.6	0.0	0.0	0.0	19.2	0.0	0.0	65.4	L
**8**	0.0	0.7	0.0	0.0	0.0	0.0	0.0	8.6	0.0	0.0	0.0	10.8	0.0	0.0	79.9	L
**40**	0.0	0.9	0.0	0.0	45.0	0.0	0.0	13.5	0.0	0.0	0.9	28.8	0.0	0.0	10.8	M
**32**	0.0	35.6	1.8	0.0	0.0	0.0	3.6	47.7	0.0	0.0	0.0	0.0	0.0	0.0	11.3	M
**37**	0.0	0.6	11.2	0.0	0.0	0.0	47.3	13.6	0.0	0.0	0.0	0.0	0.0	0.0	27.2	M
**38**	0.0	35.6	0.0	0.0	0.0	0.0	0.0	9.6	0.0	0.0	0.0	1.4	0.0	0.0	53.4	L
**35**	0.0	1.0	40.8	0.0	33.0	0.0	0.0	2.9	0.0	0.0	0.0	1.0	0.0	0.0	21.4	H
**16**	0.0	7.7	12.0	0.0	33.6	0.0	2.6	3.3	0.0	0.0	0.0	9.1	0.0	0.0	31.8	H
**36**	0.0	0.0	0.0	0.0	0.0	8.8	0.0	0.0	0.0	0.7	0.0	0.0	14.7	0.0	75.7	H
**4**	0.0	0.0	0.0	0.0	0.0	0.0	0.0	0.0	0.0	0.0	0.0	0.0	3.2	1.1	95.7	L
**18**	0.0	0.0	0.0	0.0	0.0	22.4	0.0	0.0	0.0	0.0	0.0	0.0	16.3	0.0	61.2	L
**28**	0.0	0.0	0.0	0.0	0.0	2.2	0.0	0.0	0.0	16.3	0.0	0.0	28.3	0.0	53.3	L
**11**	0.0	0.0	0.0	9.7	0.0	0.0	0.0	0.0	0.0	11.1	0.0	0.0	0.0	0.0	79.2	L
**23**	0.0	0.0	0.0	10.3	0.0	0.0	0.0	0.0	2.3	18.4	0.0	0.0	0.0	0.0	69.0	L
**1**	0.2	0.0	0.0	9.7	0.0	0.5	0.0	0.0	1.8	13.1	0.4	0.0	0.0	0.0	74.3	L
**22**	0.0	0.0	0.0	10.3	0.0	0.0	0.0	0.0	1.0	10.5	0.3	0.0	0.0	0.0	77.9	L
**9**	0.2	0.0	0.0	7.4	0.0	0.0	0.0	0.0	2.1	8.3	0.0	0.0	0.1	0.0	81.9	L
**25**	0.0	0.0	0.0	6.1	0.0	0.2	0.0	0.0	0.7	17.9	0.0	0.0	0.2	0.0	74.9	L
**24**	0.0	0.0	0.0	0.0	0.0	0.0	0.0	0.0	0.0	32.1	0.0	0.0	0.0	0.0	67.9	L
**5**	0.0	0.0	0.0	0.0	0.0	0.0	0.0	0.0	0.0	0.0	0.0	0.0	0.0	89.5	10.5	H
**13**	0.0	0.0	0.0	0.0	0.0	7.9	0.0	0.0	0.0	2.6	0.0	0.0	31.6	0.0	57.9	H
**14**	0.0	0.0	0.0	0.0	0.0	16.0	0.0	0.0	0.0	0.0	0.0	0.7	16.0	0.0	67.4	H
**17**	0.0	0.0	0.0	0.0	0.0	5.7	0.0	0.0	0.0	5.7	0.0	0.0	85.7	0.0	2.9	H
**3**	0.0	0.0	0.0	0.0	0.0	0.0	0.0	0.0	6.3	68.9	0.0	0.0	0.0	0.0	24.7	H
**19**	0.0	0.0	0.0	6.6	0.0	0.0	0.0	0.0	3.4	28.1	0.0	0.3	0.6	0.0	60.9	M
**31**	0.0	0.0	0.0	21.7	0.0	8.7	0.0	0.0	26.1	4.3	0.0	0.0	0.0	0.0	39.1	M

*Sequence types not present at greater than a 25% threshold in any of the 40 virulence groups were combined into the ST-Other group. Values for sequence type presented as percentages. VG column header describes the Virulence Group.
